# Evaluation of prognostic inflammatory and systemic inflammatory response indices in auxiliary diagnosis of bacteria-negative pulmonary tuberculosis: A diagnostic accuracy study

**DOI:** 10.1097/MD.0000000000033372

**Published:** 2023-03-24

**Authors:** Bofeng Chai, Dan Wu, Na Fu, Ping Huang, Youlu Shen, Yuhong Li, Yinghong Wang

**Affiliations:** a Qinghai University, Xining, Qinghai, China; b Qinghai University Affiliated Hospital, Xining, Qinghai, China; c Lanzhou University, Lanzhou, China; d Minle County People’s Hospital, Gansu, Zhangye, China.

**Keywords:** bacteria-negative pulmonary TB, diagnostic, prognostic inflammatory index (PII), systemic inflammatory response index (SIRI)

## Abstract

Although molecular biology has made great progress in recent years, the detection rate of mycobacterium tuberculosis (MTB) is still not ideal. This study aimed to evaluate the role of prognostic inflammatory index (PII) and systemic inflammatory response index (SIRI) in the auxiliary diagnosis of bacteria-negative pulmonary tuberculosis (TB). Sixty patients diagnosed with bacteria-negative pulmonary TB at the Affiliated Hospital of Qinghai University between October 2019 and September 2022 were randomly selected as the case group, and seventy patients with nontuberculous pulmonary infection in the same department of the same hospital during the same period were randomly selected as the control group. Baseline data and values of erythrocyte sedimentation rate (ESR), lymphocyte count (LY), neutrophil count (NE), monocyte count (MO), albumin (ALB), prealbumin (PA), C-reactive protein (CRP), fibrinogen (FIB), neutrophil-to-lymphocyte ratio (NLR), PII, and SIRI were compared between the 2 groups. Receiver operating characteristic (ROC) curves were used to evaluate the diagnostic efficacy of PII and SIRI in the diagnosis of bacteria-negative pulmonary TB. No significant differences were found between the 2 groups in terms of sex and age (*P >* .05); however significant differences were observed in relation to body mass index (BMI), ESR, LY, NE, MO, ALB, PA, CRP, FIB, NLR, PII, and SIRI (*P* < .05). ROC curve analysis showed that area under curve (AUC) value {0.84 [95% CI (0.77, 0.90)]} and specificity {82.86% [95% CI (72.0, 90.8)]} of PII were the highest, while the sensitivity {86.67 [95% CI (75.4, 94.1)]} of NLR + PII was the highest. Pairwise comparison of the 7 indicators of ROC curve was performed, and only the diagnostic efficiency of NLR and NLR + PII was statistically significant (*Z* = 2.36, *P* = .02 < .05). NLR, PII, SIRI, pairwise combinations, and NLR + PII + SIRI showed auxiliary diagnostic values for bacteria-negative pulmonary TB, among which PII had the highest diagnostic value and specificity, while NLR + PII had the highest sensitivity.

## 1. Introduction

According to a report, consolidated data submitted to the World Health Organization by 197 countries and territories covering more than 99% of the world population has shown that: The decline in the incidence of tuberculosis (TB) has halted and the number of TB-related deaths has increased for the first time in 9 years.^[1]^ The morbidity rate of TB in China in the year 2019 was 8.4%.^[2]^ In 2019, the diagnosis of pulmonary TB was bacteriologically confirmed in only 57% patients in those 197 countries.^[2]^ Positive culture of mycobacterium tuberculosis (MTB) is the gold standard for diagnosis of pulmonary TB.^[3]^ In 2019, only 45% of pulmonary TB patients in China were diagnosed as MTB-positive, while the remaining 55% pulmonary TB patients were MTB-negative.^[4]^ Due to lack of specific clinical manifestations of bacteria-negative pulmonary TB, it is often difficult to distinguish this entity from nontuberculous pulmonary infection. It can only be comprehensively diagnosed based on the exclusion of other diseases, combined with nonspecific medical history, imaging, and laboratory results of the patient, which begets a lot of inconvenience in the patients’ treatment As a result, the disability and mortality rate of bacteria-negative TB patients remains higher than those of culture-positive patients, which affects their quality of life,^[5]^ and results in economic and mental burden on the family and society. At present, there is an urgent need to increase the diagnostic rate of bacteria-negative pulmonary TB.

Although molecular biology has made great progress in recent years, the detection rate of MTB is still not ideal.^[3]^ Studies have shown that prognostic inflammatory index (PII) and systemic inflammatory response index (SIRI) are new indicators for evaluating systemic inflammation, nutrition, and immune status; which have been used in the diagnosis and prognosis of cancer and infectious diseases, such as breast cancer, gastric cancer, rectal cancer, and infective endocarditis.^[6–9]^ PII and SIRI are calculated according to the laboratory indicators of patients during hospitalization, which has the added advantages of simplicity and convenience.^[6,10]^ Pathological changes in pulmonary TB mainly include inflammatory exudation, hyperplasia, and caseous necrosis.^[11]^ To the best of our knowledge, no study has utilized the efficacy of PII and SIRI in the diagnosis of bacteria-negative pulmonary TB. Thus, this study aimed to explore the auxiliary value of PII and SIRI in the diagnosis of bacteria-negative pulmonary TB.

## 2. Methods

### 2.1. Study population

Sixty patients diagnosed with bacteria-negative pulmonary TB at the Qinghai University Affiliated Hospital between October 2019 and September 2022 were randomly selected as the case group, and seventy patients with nontuberculous pulmonary infection in the same department of the same hospital during the same period were randomly selected as the control group. All patients enrolled in the study were diagnosed based on inclusion and exclusion criteria by 2 investigators.

### 2.2. Ethical review

Our study had been checked and approved by the Ethics Committee of Qinghai University Affiliated Hospital.

### 2.3. Inclusion and exclusion criteria

Inclusion criteria were as follows: inpatients diagnosed with bacteria-negative TB (in accordance with the People Republic of China Health Industry Standards “diagnosis standard of pulmonary tuberculosis (WS288-2017)”^[12]^ and “Tuberculosis classification (WS196-2017)”^[13]^ issued in 2018); age ≥ 18 years; complete clinical data and standard first examination at the time of admission.

The exclusion criteria were as follows: chronic diseases such as hypertension, diabetes, coronary heart disease, and chronic obstructive pulmonary disease; extrapulmonary TB; complications with tumor and autoimmune diseases; hepatitis B or C; severe infection; and absence of sputum culture.

### 2.4. Study indices

Baseline data including sex, age, body mass index (BMI); and laboratory data including neutrophil count (NE), lymphocyte count (LY), monocyte count (MO), C-reactive protein (CRP), prealbumin (PA), albumin (ALB), fibrinogen (FIB), and erythrocyte sedimentation rate (ESR) were collected. The detection method of laboratory data was as follows: we took fasting venous blood from the patient in the morning, separated the serum, and detected by immunoturbidimetry. Further, neutrophil-lymphocyte ratio, PII, and SIRI were calculated using the following formulae.

NLR = neutrophils/lymphocytes; PII = CRP × NLR/ALB; SIRI = NLR × MO.

### 2.5. Statistical analysis

The final data were processed using SPSS 27.0 (IBM Corp., Armonk, NY) and MedCalc 20.1 (MedCalc Software Ltd., Ostend, Belgium) software. Measurement data with normal distribution were expressed as mean ± standard deviation (mean ± SD), and comparisons between groups were performed using 2-sample *t* test. Median and quartile [M (P25, P75)] were used to express skewed distribution data. Wilcoxon rank sum test was used for comparison between groups. Dichotomous data were represented by constituent ratio, and Chi-square test was used for comparison. Receiver operating characteristic (ROC) curve was used to determine the diagnostic and optimal cutoff values of NLR, PII, SIRI, pairwise combinations, and NLR + PII + SIRI for bacteria-negative pulmonary TB. Statistical significance was defined as *P* < .05.

## 3. Results

### 3.1. Baseline data

A total of 130 patients with confirmed bacteria-negative pulmonary TB or nontuberculous pulmonary infection were included in this study, of which 45.38% were males with age ranging between 18 and 81 years. In the control group of seventy cases of nontuberculous pulmonary infection, males aged 18 to 75 years accounted for 51.43%, with a median age of 40 (26.50, 53.25) years. The case group included 60 cases of bacteria-negative pulmonary TB, with 38.33% male, aged 18 to 81 years, with a median age of 42.5 (24.00, 56.00) years. Statistically significant differences were observed between the 2 groups in terms of ESR, BMI, LY, NE, MO, ALB, PA, CRP, FIB, NLR, PII, and SIRI (*P* < .05; Table 1).

**Table 1:** Comparison of general data and laboratory indicators between the 2 groups.

Group index	Control group	Case group	*t*/*X*^2^/*Z*	*P* value
	n = 70	n = 60		
Male (n, %)	36 (51.43)	23 (38.33)	2.26	.14
Age (M (P_25_, P_75_))	40 (26.50, 53.25)	42.5 (24.00, 56.00)	−0.18	.86
BMI (kg/m^2^, mean ± SD)	23.05 ± 3.75	21.56 ± 3.66	2.27	.03*
LY (10^9^/L, mean ± SD)	1.84 ± 0.62	1.23 ± 0.37	6.82	<.001*
NE (10^9^/L, M (P_25_, P_75_))	3.58 (2.84, 4.46)	4.12 (3.17, 5.23)	−2.20	.03*
MO (10^9^/L, M (P_25_, P_75_))	0.40 (0.33, 0.48)	0.48 (0.37, 0.61)	−3.28	.001*
ALB (g/L, mean ± SD)	42.82 ± 3.70	38.87 ± 3.92	5.90	<.001*
PA (mg/L, mean ± SD)	274.81 ± 76.20	210.56 ± 61.74	5.23	<.001*
CRP (mg/L, M (P_25_, P_75_))	0.90 (0.30, 3.80)	8.50 (3.35, 24.4)	−6.04	<.001*
FIB (g/L, M (P_25_, P_75_))	2.64 (2.20, 3.04)	3.61 (2.89, 4.60)	−5.89	<.001*
ESR (mm/h, M (P_25_, P_75_))	5.50 (2.00, 10.00)	16.5 (8.50, 33.75)	−6.19	<.001*
NLR (M (P_25_, P_75_))	2.02 (1.40, 2.75)	3.23 (2.65, 4.53)	−6.09	<.001*
PII (M (P_25_, P_75_))	0.04 (0.02, 0.18)	0.60 (0.24, 2.68)	−6.72	<.001*
SIRI (M (P_25_, P_75_))	0.81 (0.59, 0.97)	1.64 (1.04, 2.44)	−6.31	<.001*

ALB = albumin, BMI = body mass index, CRP = C-reactive protein, ESR = erythrocyte sedimentation rate, FIB = fibrinogen, LY = lymphocyte count, MO = monocyte count, NE = neutrophil count, NLR = neutrophil-to-lymphocyte ratio, PA = prealbumin, PII = prognostic inflammatory index, SIRI = systemic inflammatory response index.

* Statistically significant.

### 3.2. ROC curve analysis

To further explore the efficacy of NLR, PII, SIRI, pairwise combinations, and NLR + PII + SIRI in the diagnosis of bacteria-negative pulmonary TB, the case and control groups were used as positive and negative samples, respectively, a regression equation was constructed, and a diagnostic analysis model of ROC curve was established.

**Figure 1.**
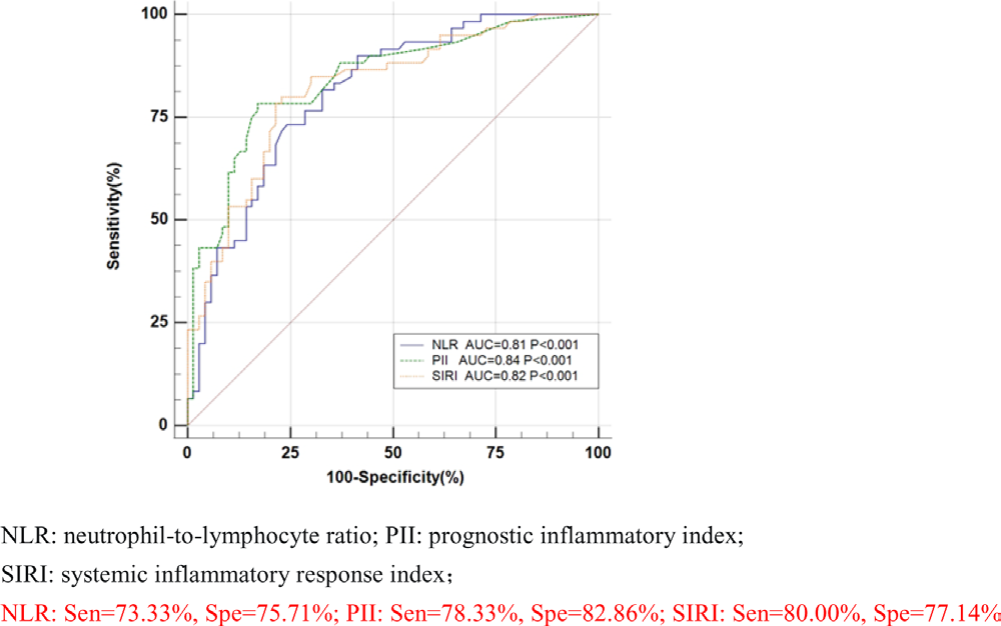
Receiver operating characteristic (ROC) curves of NLR, PII and SIRI. NLR = neutrophil-to-lymphocyte ratio, PII = prognostic inflammatory index, SIRI = systemic inflammatory response index; NLR: Sen = 73.33%, Spe = 75.71%; PII: Sen = 78.33%, Spe = 82.86%; SIRI: Sen = 80.00%, Spe = 77.14%.

**Figure 2.**
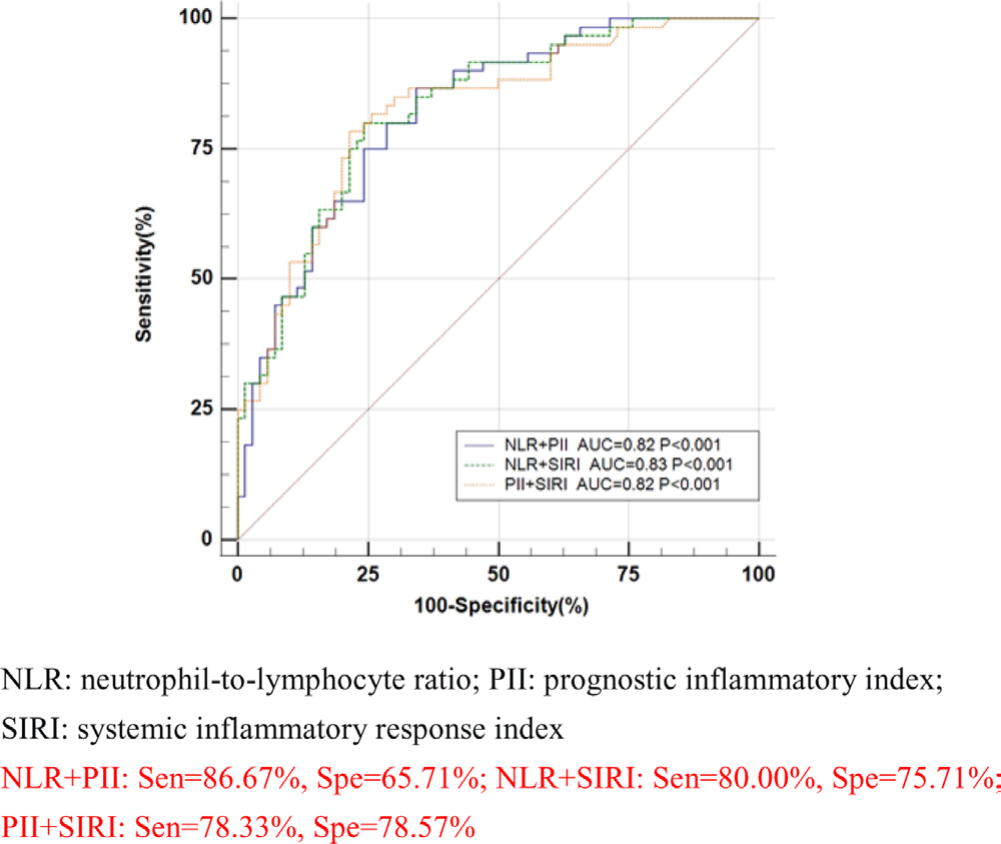
Receiver operating characteristic (ROC) curves of pairwise combined. NLR = neutrophil-to-lymphocyte ratio, PII = prognostic inflammatory index, SIRI = systemic inflammatory response index; NLR + PII: Sen = 86.67%, Spe = 65.71%; NLR + SIRI: Sen = 80.00%, Spe = 75.71%; PII + SIRI: Sen = 78.33%, Spe = 78.57%.

**Figure 3.**
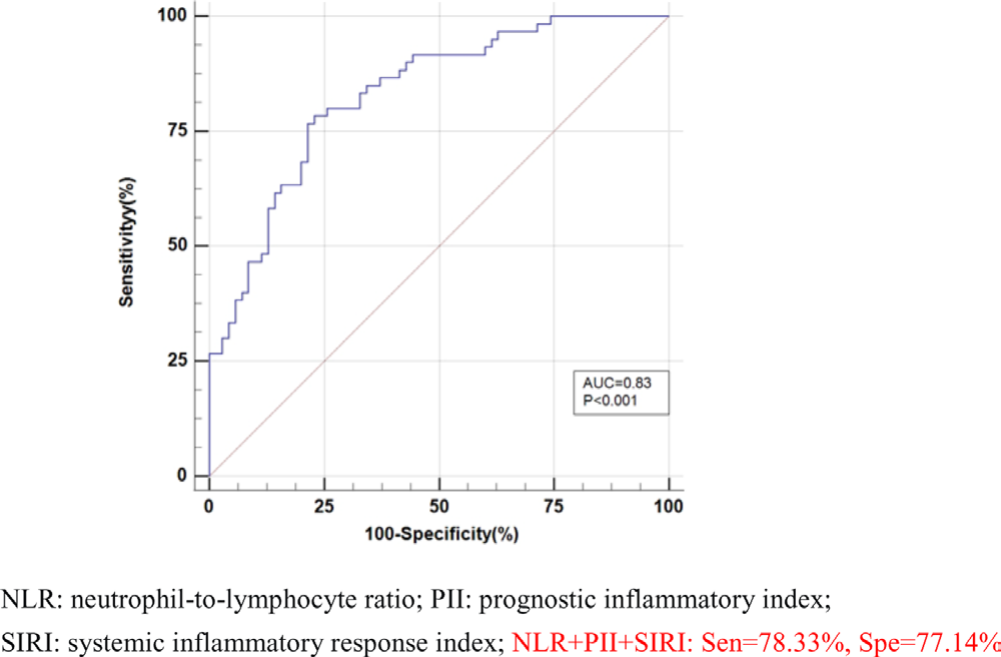
Receiver operating characteristic (ROC) curve of NLR + PII + SIRI. NLR = neutrophil-to-lymphocyte ratio, PII = prognostic inflammatory index, SIRI = systemic inflammatory response index; NLR + PII + SIRI: Sen = 78.33%, Spe = 77.14%.

The results showed that area under curve (AUC) value, sensitivity and specificity were respectively 0.84 [95%CI (0.77, 0.90)], 78.33%[95%CI (65.8, 87.9)] and 82.86%[95%CI (72.0, 90.8)] of PII; 0.82 [95%CI (0.74, 0.88)], 80.00%[95%CI (67.7, 89.2)] and 77.14%[95%CI (65.6, 86.3)] of SIRI; 0.81, 73.33% and 75.71% of NLR; 0.82, 86.67% and 65.71% of NLR + PII; 0.83, 80.00% and 75.71% of NLR + SIRI; 0.82,78.33% and 78.57% of PII + SIRI; 0.83, 78.33% and 78.57% of NLR + PII + SIRI; and the AUC value and specificity of PII were the highest, while the sensitivity of NLR + PII had the maximum value (Table 2). ROC curves were constructed for the 7 indicators, as shown in Figures 1, 2, and 3. Pairwise comparison showed that out of all the indicators, the diagnostic efficiency of only NLR and NLR + PII demonstrated significant differences (Z = 2.36, *P* = .02 < .05) (Table 3).

**Table 2:** Receiver operating characteristic curves (ROC) analysis results of the 7 indices.

Index	Cutoff value	AUC	95% CI		Sen%	95% CI		Spe%	95% CI	
			L	U		L	U		L	U
NLR	2.74	0.81	0.73	0.87	73.33	60.3	83.9	75.71	64.0	85.2
PII	0.20	0.84*	0.77	0.90	78.33	65.8	87.9	82.86†	72.0	90.8
SIRI	0.97	0.82	0.74	0.88	80.00	67.7	89.2	77.14	65.6	86.3
NLR + PII	–	0.82	0.75	0.88	86.67‡	75.4	94.1	65.71	53.4	76.7
NLR + SIRI	–	0.83	0.75	0.89	80.00	67.7	89.2	75.71	64.0	85.2
PII + SIRI	–	0.82	0.75	0.89	78.33	65.8	87.9	78.57	67.1	87.5
NLR + PII + SIRI	–	0.83	0.76	0.89	78.33	65.8	87.9	77.14	65.6	86.3

AUC = area under curve, CI = confidence Interval, *L* = lower limit, NLR = neutrophil-to-lymphocyte ratio, PII = prognostic inflammatory index, SIRI = systemic inflammatory response index, *U* = upper limit.

* AUC had the maximum value.

† Specificity had the maximum value.

‡ Specificity had the maximum value.

**Table 3:** Pairwise comparison results of the 7 indicators (*Z* value, *P* value).

PII + SIRI	*Z =* 0.69					
	*P =* .49					
NLR + SIRI	*Z =* 1.71 *P =* .09	*Z =* 0.36 *P =* .72				
NLR + PII	*Z =* 0.65 *P =* .52	*Z =* 0.10 *P =* .92	*Z =* 0.41 *P =* .68			
SIRI	*Z =* 0.91 *P =* .36	*Z =* 0.74 *P =* .46	*Z =* 0.61 *P =* .54	*Z =* 0.02 *P =* .99		
PII	*Z =* 0.32 *P =* .75	*Z =* 0.53 *P =* .60	*Z =* 0.40 *P =* .69	*Z =* 0.55 *P =* .58	*Z =* 0.57 *P =* .57	
NLR	*Z =* 1.38 *P =* .17	*Z =* 0.59 *P =* .55	*Z =* 1.17 *P =* .24	*Z =* 2.36 *P =* .02*	*Z =* 0.47 *P =* .64	*Z =* 0.80 *P =* .42
	NLR + PII + SIRI	PII + SIRI	NLR + SIRI	NLR + PII	SIRI	PII

NLR = neutrophil-to-lymphocyte ratio, PII = prognostic inflammatory index, SIRI = systemic inflammatory response index.

* Statistically significant.

## 4. Discussion

As a chronic wasting infectious disease, TB is mainly transmitted through air by the MTB complex.^[14]^ The gold standard for diagnosis of TB is a positive MTB culture.^[3]^ However, due to the slow growth and long culture cycle of MTB, the positive culture rate is not ideal,^[15]^ which delays diagnosis and treatment. In recent years, new techniques have been used in the diagnosis of TB, such as MTBDRplus, loop-mediated isothermal amplification (LAMP), line probe detection (LPA), GeneXpert, and whole genome sequencing (WGS).^[14]^ In addition, technologies like portable volatile organic compound (VOC) technology^[16]^ and instant eNose technology,^[17]^ which are in the research stage can also be used; however, they have not been popularized among hospital laboratories or patients due to cost factor or immature technology.

Once MTB enters the body, it initiates inherent and adaptive immunity for resistance.^[18]^ Adaptive immunity caused by MTB is mainly a T cell-mediated immune response; of which, CD4 + T lymphocytes play an important role in antiTB immunity.^[19]^ It is important to further inhibit or kill MTB, which has a decisive impact on the occurrence, development, and outcome of TB.^[18]^ In recent years, percentage and absolute count of peripheral blood lymphocytes has widely been used in the diagnosis of TB and predicting its severity and prognosis.^[20,21]^ PII and SIRI, which are derived by simple calculation from LY, NE, MO, CRP, and ALB, can reflect both the immune and systemic nutritional status of patients.^[6,10]^ To the best of our knowledge, there are no domestic or international relevant studies on the auxiliary diagnostic value of PII and SIRI for pulmonary TB. The results of this study showed that LY, ALB, and PA were significantly lower in the case group as compared to the control group (*P <* .05); while NE, MO, CRP, FIB, ESR, NLR, PII, and SIRI were significantly higher in the case group (*P <* .05). This was consistent with the findings of *Cázares-Sosa FR* et al^[22]^ and *An HR* et al^[23]^ Reduction in LY count occurs since T lymphocytes limit MTB growth by cracking the MTB-infected cells.^[24]^ Increased NE is associated with decreased LY.^[25]^ Increased NE can protect the organism from MTB infection,^[26]^ but large numbers of neutrophils can destroy lung tissue through their collective oxidative burst action and result in formation of lung cavities^[27,28]^ and pulmonary cavities^[28]^ and is positively associated with disease severity and mortality.^[29,30]^ *Castaño D* et al reported that the percentage of intermediate and nonclassical monocytes increased after TB infection, while the percentage of classic monocytes decreased; however, increase and decrease in CD16 + MOs were noted after antiTB treatment.^[31–33]^ They also found that intermediate monocytes played a significant role in T-lymphocyte activation, proliferation, and antigen presentation.^[31]^ ALB and PA reflect human nutritional and chronic inflammation status^[32]^ and are involved in multiple physiological functions. PA, also known as transthyroxine protein, is more sensitive than ALB in assessing the nutritional status of patients.^[34]^ In the study by *Yi Li* et al,^[35]^ 64.41% of 295 inpatients with TB were at nutritional risk. They reported significant differences between the case and control groups in terms of age, combined diseases, BMI, and ALB level (*P* < .05). Significant differences were also noted in the incidence of complications and length of hospital stay (*P <* .05), among which nosocomial secondary infections (43.05%) and abnormal liver function (13.56%) were the most common complications.^[35]^ In addition, TB often leads to decreased gastrointestinal function, which results in inadequate nutrient intake and reduced anabolic metabolism.^[36]^ Protein in the body can be metabolized by MTB, thus, symptoms of malnutrition occur, which impairs the body nutritional and energy status.^[36]^

In this study, ROC curves were constructed using 3 separate indicators of NLR, PII, and SIRI; pairwise combination; and indicators of NLR + PII + SIRI. The results showed that AUC value of PII was the largest (0.84) and had the highest specificity (82.86%), While PII + NLR had the highest sensitivity (86.67%). Pairwise comparison of all indicators of ROC curve showed that the diagnostic efficiency of only NLR and NLR + PII were statistically significant (*Z* = 2.36, *P* = .02 < .05).

This study had some limitations. It was a retrospective case-control study, and thus, was susceptible to selection, information, confounding bias, and causal relationship between the indices, bacteria-negative TB, and severity could not be clarified. Moreover, this was a single-center, small-sample study. Henceforth, it is necessary to carry out multi-center, large-sample studies in the future to prove the clinical application value of these indicators.

In conclusion, PII and SIRI were demonstrated to have clinical application value in differentiating patients with nontuberculous pulmonary infection and tuberculous pulmonary infection. Based on the combination of patient symptoms and medical history, PII had the highest diagnostic value. Reduction of time and cost factors for the diagnosis of bacteria-negative TB patients would aid in subsequent treatment and improvement of the quality of life of these patients.

## Acknowledgments

We would like to express our gratitude to all patients.

## Author contributions

**Conceptualization:** Bofeng Chai, Dan Wu, Youlu Shen, Yuhong Li.

**Data curation:** Bofeng Chai, Ping Huang, Yinghong Wang.

**Formal analysis:** Bofeng Chai, Na Fu, Ping Huang.

**Investigation:** Bofeng Chai, Dan Wu, Ping Huang.

**Methodology:** Bofeng Chai, Dan Wu, Yinghong Wang.

**Project administration:** Bofeng Chai.

**Resources:** Bofeng Chai, Dan Wu, Na Fu.

**Software:** Bofeng Chai.

**Supervision:** Youlu Shen, Yuhong Li.

**Validation:** Bofeng Chai.

**Visualization:** Bofeng Chai.

**Writing – original draft:** Bofeng Chai.
